# Preliminary results with Habib’s procedure

**DOI:** 10.1186/1471-2318-11-S1-A32

**Published:** 2011-08-24

**Authors:** R Mastromarino, S D’Angelo, A Saglioccolo, G Rizzo, V Tammaro, N Carlomagno, A Renda

**Affiliations:** 1 Department of Surgical, Anesthesiology-rianimative and Emergency Sciences, Federico II University, Naples, Italy

## Background

In recent years liver resection has increasingly been performed. Bleeding and hepatic acute failure, secondary to vascular inflow occlusion (Pringle’s manoeuvre), remain significant complications; these problems seem to be higher in older patients and in those with cirrhosis.

Several surgical tools such as CUSA, Harmonic scalpel, Bipolar scissors, Ligasure diathermy, have been developed to decrease blood loss. Another device, based on radiofrequency, was proposed by Habib.1

The aim of our study was to evaluate Habib’s procedure and its influence in the outcomes of patients.

## Materials and methods

From January 2009 to November 2010 we observed 11 patients with colo-rectal Cancer Heaptic metastases (ME) and 3 HCC patients; they were aged 71-84 y.o. ( median age 75 years), m:f ratio = 1. 3. In all cases Habib’s device and intraoperative US were routinely adopted. 2 Several parameters have been analyzed: age, sex, smoke abuse, comorbidity, ASA score, Child score, HBV+, HCV+, operation length, intraoperative bleeding, postoperative outcome, bowel motility, drainages output, hospital stay.

## Results

We performed: metastasectomy (17 in 11 patients), wedge resection, (3 pts). We never used Pringle’s manoeuvre. Mean intraoperative blood loss was 150 ml (range 100-350 cc) and only one patient required a blood transfusion. The mean operation time was 90 min. Postoperative outcome was accetable in all cases. Bowel motility start at 48h (range 24-36h). Mean drainage output was 150 cc at 24h and 80cc at 48h. There were no major systemic complications but one patient postoperatively suffered hepato-renal failure at the intensive care unit. Patients were discharged on the fifth day (range 4- 12 days).

## Discussion

Habib’s device allowed us to perform minor or non-anatomical hepatic resections without Pringle’s manoeuvre and with minimal blood loss.

In our experience postoperative hemorrhage and biliary leak were minimal, supporting the effectiveness of the device.

Along with a low rate of resection specific complications, the rate of overall postoperative complication in this series was low.

## Conclusion

Our results, despite the numbers, have been encouraging. It is our opinion that Habib’s device allows liver resections to be carried out with minimal blood loss and low morbidity and mortality rates.
